# Incidence and determinants of perinatal mortality among women with obstructed labour in eastern Uganda: a prospective cohort study

**DOI:** 10.1186/s40748-021-00133-7

**Published:** 2021-07-15

**Authors:** Milton W. Musaba, Grace Ndeezi, Justus K. Barageine, Andrew D. Weeks, Julius N. Wandabwa, David Mukunya, Paul Waako, Beatrice Odongkara, Agnes Arach, Kenneth Tulya-muhika Mugabe, Agnes Kasede Napyo, Victoria Nankabirwa, James K. Tumwine

**Affiliations:** 1 Department of Obstetrics and Gynaecology, Mbale Regional Referral and Teaching Hospital, Mbale, Uganda; 2 Department of Obstetrics and Gynaecology, Busitema University Faculty of Health Sciences, Mbale, Uganda; 3 Department of Paediatrics and Child Health, School of Medicine, Makerere University College of Health Sciences, Kampala, Uganda; 4 Department of Obstetrics & Gynaecology, School of Medicine, Makerere University College of Health Sciences, Kampala, Uganda; 5 Sanyu Research Unit, University of Liverpool/Liverpool Women’s Hospital, Liverpool, UK; 6 Department of Public and Community Health, Busitema University Faculty of Health Sciences, Mbale, Uganda; 7 Department of Pharmacology and Therapeutics, Busitema University Faculty of Health Sciences, Mbale, Uganda; 8 Department of Paediatrics and Child Health, Gulu University, Gulu, Uganda; 9 Department of Nursing and Midwifery, Lira University, Lira, Uganda; 10 Department of Public and Community Health, Busitema University Faculty of Health Sciences, Mbale, Uganda; 11 Department of Epidemiology and Biostatistics, School of Public Health, Makerere University College of Health Sciences, Kampala, Uganda; 12 Centre for Intervention Science and Maternal Child health (CISMAC), Center for International Health, University of Bergen, Bergen, Norway

**Keywords:** Obstructed labour, Perinatal death, Determinants, Lactate

## Abstract

**Background:**

In Uganda, the incidence and determinants of perinatal death in obstructed labour are not well documented. We determined the incidence and determinants of perinatal mortality among women with obstructed labour in Eastern Uganda.

**Methods:**

Between July 2018 and September 2019, 584 with obstructed labour were recruited and followed up to the 7th day postnatal. Information on maternal characteristics, obstetric factors and laboratory parameters was collected. Each patient received the standard perioperative care. We used a generalized linear model for the Poisson family, with a log link and robust variance estimation to determine the association between the exposure variables and perinatal death.

**Results:**

Of the 623 women diagnosed with obstructed labour, 584 met the eligibility criteria. There were 24 fresh still births (FSB) and 32 early neonatal deaths (ENND) giving an FSB rate of 43.8 (95% CI 28.3–64.4) deaths per 1000 total births; early neonatal death rate of 58.4 (95% CI 40.3–81.4) deaths per 1000 and an overall perinatal mortality rate of 102.2 (95% CI 79.4–130.6) deaths in the first 7 days of life. A mother being referred in active labour adjusted risk ratio of 2.84 (95% CI: 1.35–5.96) and having high blood lactate levels at recruitment adjusted risk ratio 2.71 (95% CI: 1.26–4.24) were the determinants of perinatal deaths.

**Conclusions:**

The incidence of perinatal death was four times the regional and national average. Babies to women referred in active labour and those with high maternal blood lactate were more likely to die.

## Background

Uganda’s perinatal mortality has stagnated at 40 deaths per 1000 pregnancies over the last decade [1]. This is three times the Sustainable Development Goal (SDG) and Every Newborn Action Plan (ENAP) target of ≤12/1000 neonatal deaths by 2030 [2]. Over the same period, the utilization of maternity services has generally improved. According to the 2016 Uganda Demographic and Health Survey (UDHS) report, 1st ANC attendance was at 95 and 65% of births were delivered by a skilled birth attendant [1]. Despite all these achievements, about half of the reported perinatal deaths are attributed to obstructed labour and 90% of them are caused by intrapartum birth asphyxia [3]. Strong and frequent uterine contractions in obstructed labour lead to intrapartum hypoxia. This, in turn sets a fetus on a pathway to asphyxia, acidosis, neuronal injury, long term morbidity and or death, if the obstruction is not relieved quickly [4, 5].

Intrapartum surveillance using the partogram is one of the key interventions aimed at reducing perinatal morbidity and mortality through early detection of labour complications [6]. However, in several resource limited settings, its uptake and subsequent impact has been minimal because of various reasons [7]. Including the fact that, maternity care is often characterized by a mismatch between high patient volumes and low staffing levels [7]. The risk factors for perinatal death are well documented, they are mostly related to limited or no access to quality maternity health care services [8–10]. Among high-risk obstetric populations such as obstructed labour, epidemiological information about the incidence and risk factors for perinatal death is scarce. Yet for Uganda to achieve the SDG or ENAP targets, focused interventions among such high-risk obstetric populations need to be implemented.

Compared to normal labour, obstructed labour is associated with higher levels of lactate [11, 12], and derangements in various laboratory parameters such as electrolytes, liver and renal function [13]. In several high resource settings, measurement of lactate in amniotic fluid, fetal scalp and arterial cord blood is used in clinical practice as a predictor of fetal outcomes [4, 14, 15]. Lactate is a byproduct of fetal maternal metabolism and its levels vary throughout the process of labour in response to intermittent hypoxia [11, 16]. A recent systematic review concluded that lactate levels measured during the intrapartum period are a good predictor of fetal and maternal outcomes in prolonged and difficult labour [5]. Lactate can be easily measured at the bedside using a low cost, low maintenance, battery operated and easy to use point of care devices [5, 17]. This makes lactate measurement an attractive option that can be adopted to compliment the current standard of care for intrapartum fetal and maternal surveillance. Studies from high resource settings report an association between high fetal blood lactate and neonatal morbidity among women with labour dystocia and not obstructed labour. We hypothesized that high maternal lactate among women with obstructed labour was associated with a high perinatal mortality rate.

## Methods

### Study setting

The study was conducted in the labour ward of Mbale regional referral hospital between July 2018 and September 2019 [18]. This hospital has a catchment population of about four million people from 14 districts in and around the Elgon subregion. Mbale hospital is the main referral center for the four district hospitals and 10 health sub-districts in and around this sub-region Annually, about 5% (600) of the women that deliver in Mbale hospital are diagnosed with obstructed labour [19]. The annual caesarean section rate is 35%. The labour ward has a dedicated emergency obstetric care theatre with one operating bed. The department is staffed with two Obstetricians, two Medical Officers and 21 Midwives. The neonatal unit is housed within the department of obstetrics and gynaecology next the labour suite. This unit is run by a Neonatologist.

### Study design

This was a prospective cohort study in which women with obstructed labour were recruited at the time of diagnosis and the mother-baby pairs were followed up to the 7th postnatal day.

### Study participants

Women diagnosed with obstructed labour at Mbale regional hospital formed the study population. All women admitted to the labour ward in active labour were the source population.

### Study procedure

During the study period, all women diagnosed with obstructed labour by an Obstetrician or Medical Officer on duty were screened with the aim of enrolling eligible participants into an ongoing trial PACTR201805003364421 [18]. Midwives in the delivery suite were sensitized about the ongoing project and informed the study team of all potential participants diagnosed with obstructed labour. Two research assistants were available throughout the day and night with the aim of recruiting eligible women with obstructed labour [18, 20].

All of them received the Ministry of Health recommended standard preoperative care for patients with OL. This package includes; antibiotic prophylaxis, intravenous fluid replacement (1.5 L of normal saline), bladder drainage and lying in the left-lateral position as they were being prepared for emergency caesarean section [21]. Five mls of venous blood was drawn from the antecubital fossa of each patient for a complete blood count, blood grouping and cross matching which are part of the standard care. In addition, electrolytes, liver function tests and lactate levels were measured as part of the extra procedures for the study. Maternal blood lactate levels were measured using a bedside point of care device at recruitment, at 1 h and from the myometrium at caserean section as detailed in the published study protocol [18]. Two hundred and thirty-eight (238/548) participants randomised into the active arm of the trial received a single bolus infusion of 4.2 g of sodium bicarbonate in the preoperative period in addition to the standard of care.

### Sample size and sampling

We included 548 newborns to women diagnosed with obstructed labour. This was deemed adequate to detect a 30% difference in the rate of perinatal death between those mothers with high and normal maternal blood lactate at the time of recruitment. We assumed that the incidence of perinatal deaths in the unexposed group was 41 per 1000 total births [22]. We further assumed a power of 80 and 95% level of confidence. Eligible participants were consecutively enrolled from July 2018 to September 2019.

### Eligibility criteria

We included all eligible patients with OL carrying live, singleton, term pregnancies (≥37 gestation age) in cephalic presentation at the time of diagnosis. Whereas those diagnosed with both obstructed labour and other obstetric emergencies such as (antepartum hemorrhage, Pre-eclampsia and eclampsia (defined as elevated blood pressure of at least 140/90 mmHg, urine protein of at least 2+, any of the danger signs and fits), premature rupture of membranes and intrauterine fetal death were excluded from this study. We also excluded those with medical comorbidities such as diabetes mellitus, sickle cell disease because they increase the risk of morbidity and mortality as well.

### Study variables

#### Outcome variable

The primary outcome variable was incidence of perinatal death among women with obstructed labour. Perinatal death was defined as the demise of a fetus from the time when the mother was recruited into the study up to the 7th day postnatal. Obstructed labour was diagnosed by either an Obstetrician or Medical Officer on duty using a definition of the American Association of Obstetricians and Gynaecologists (ACOG). In the first stage of labour, she should have cervical dilatation > 6 cm with ruptured membranes, adequate contractions lasting > 4 h with no change in cervical dilatation or delay in the second active stage of labour (nullipara > 2 h, multipara > 1 h) with adequate uterine contractions. In addition, any two of: the obvious signs of severe obstruction such as caput formation, severe moulding, Bandl’s ring, subconjunctival haemorrhages or an oedematous vulva [19].

#### Exposure variables

We collected data on several maternal characteristics and intrapartum factors from recruitment to discharge. The maternal characteristics included age, height, weight, parity, duration of labour, maternal lactate, hemoglobin level, history of rupture of membranes (yes vs.no), being referred from a lower health facility (yes vs. no), using tranditional medicines in labour (yes vs. no) While the fetal characteristics included birth weight, cord blood lactate and the activity, pulse, grimace, appearance, respiration (Apgar) score. Maternal blood lactate level measured at diagnosis of obstructed labour was our main exposure variable, categorized into high and normal using a cut off of 4.8 mmol/L from the NICE green top guidelines [10].

#### Data collection and management

A team of six well trained research assistant, who were also qualified midwives used an interviewer administered electronic questionnaire from enrollment up to the 7th day postnatal to collect information from the mothers. Data was collected using Open Data Kit software [23], on password protected android tablets. The research assistants were also trained to measure lactate using the Lactate Pro2 (Arkray, Japan Shiga) point of care devices by principal investigator (PI). The collected data was saved on a password protected aggregate server to which only the PI had access for conducting daily checks to ensure completeness of the uploaded questionnaires.

#### Data analysis

We used Stata version 14.0 (StataCorp; College Station, TX, USA) to analyze the data. Participant characteristics were compared across the two groups (perinatal death versus no perinatal death) and summarized as proportions for categorical data and means with standard deviations or medians with the interquartile range for continuous data. Maternal blood lactate level at baseline was categorized into high and normal using the NICE green top guideline of 4.8 mmol/L for acidemia [24], and presented as frequencies and percentages. At bivariable analysis, we used a generalized linear model for the Poisson family, with a log link and robust variance estimation to determine the association between the various exposure variables and perinatal death [25]. At multivariable analysis, intrapartum factors included in the model were determined a priori during a review of the literature on the subject. We also drew a directed acyclic graph (DAG) [26] to select variables for our final adjusted model which included: history of being referred, use of herbs during labour, history of rupture of membranes and level of lactate in maternal blood, and duration of labour details are at http://dagitty.net/mYJiolI . All variables included in the model were assessed for collinearity and considered collinear if they had a variance inflation factor greater than 10. In the case of collinearity, we retained the variable with greater biological plausibility and/or measure of association.

## Results

**Fig. 1:**
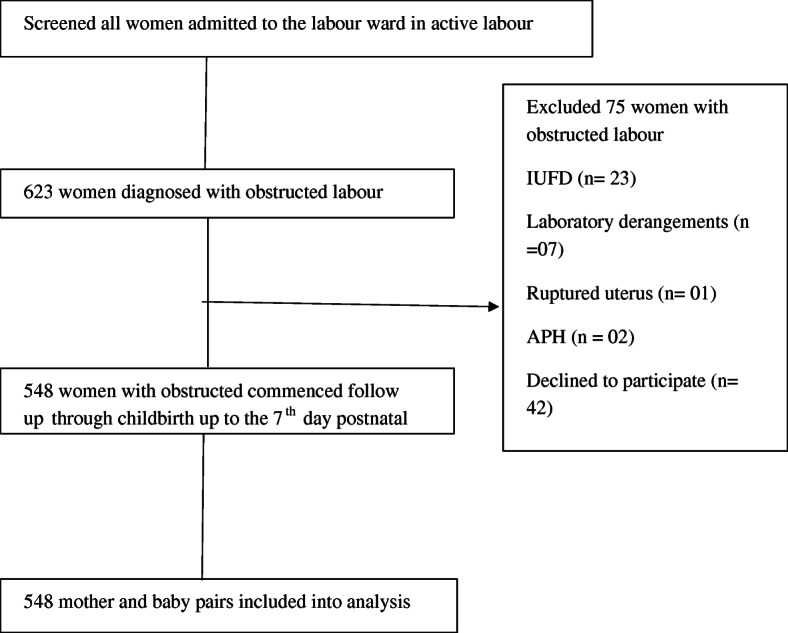
Flow chart of recruitment of women with obstructed labour in Eastern Uganda

Over a period of 13 months, 623 patients were diagnosed with obstructed labour. Of these 75 were excluded because they did not meet the study eligibility criteria. Details are in Fig. 1. Over 90% of the participants in this study delivered by emergency caserean section. A few eventually had a vaginal delivery as they were waiting to get access to theatre. Every effort was made to ensure that a delivery was achieved within 2 h of recruitment [27].

### Participant characteristics

The mean age of the participants was 24 years [(Standard Deviation (SD) 7)]. Overall, the median maternal blood lactate level was 6.6 mmol/L (3.4–12.2) and 60% (*n* = 331) of them were acidotic. Their median maternal lactate was 8.3 mmol/L (interquartile range (IQR) (5–14) among those with perinatal deaths compared to 6.3 mmol/ (3.3–12) among those with live babies. The median duration of labour was 24 (15–49) hours among those with perinatal deaths compared to 29 (20–43) hours among those with live babies. Other study characteristics are presented in Table 1.

**Table 1:** Characteristics of 548 women with obstructed labour in Mbale hospital

Variable	Perinatal death	
	Yes (***n*** = 56)	No (***n*** = 492)
**Mean maternal weight (SD)**	62 (10)	65 (12)
**Mean maternal height (SD)**	155 (15)	158 (10)
**Mean fetal heart rate on admission (SD)**	115 (55)	138 (13)
**Mean maternal age (SD)**	24 (7)	24 (6)
< 20 years (n, %)	21 (38)	133 (27)
20–35 years (n, %)	31 (55)	334 (68)
> 35 years (n, %)	4 (7)	25 (5)
**Median duration of labour, hours (IQR)**	24.4 (15.4–40.9)	28.8 (18.7–42.5)
**Gravidity** (n, %)		
Prim para	29 (52)	266 (54)
Gravida 2 to 4	17 (30)	162 (33)
Gravid 5+	10 (18)	64 (13)
**Use of traditional medicine** (n, %)		
Yes	43 (77)	272 (55)
No	13 (23)	218 (45)
**History of referral** (n, %)		
Yes	48 (86)	300 (61)
No	8 (14)	192 (39)
**History of rupture of membranes** (n, %)		
Yes	51 (91)	395 (80)
No	5 (9)	97 (20)
**Maternal venous lactate, mmol/L**		
**At base line**	8.3 (5–14)	6.3 (3.3–12)
Normal lactate (<=4.8)	12 (21)	205 (41)
High lactate (> 4.8)	44 (79)	287 (59)
**Myometrial blood lactate**^a^	**10.9 (6.4–18.2)**	**6.4 (3.4–13)**
Normal lactate (<=4.8)	16 (29)	253 (51)
High lactate (> 4.8)	40 (71)	239 (49)
**At one hour**	10.9 (6.4–18.2)	6.4 (3.4–13)
Normal lactate (<=4.8)	11 (20)	183 (37)
High lactate (> 4.8)	45 (80)	309 (63)

^a^Median and interquartile range (*N* = 490)

### Perinatal mortality

Of the 548 women with obstructed labour, there were 24 fresh still births (FSB) and 32 early neonatal deaths (ENND) giving a fresh still birth rate of 43.8 deaths per 1000 total births (95% CI = 28.3–64.4); an early neonatal death rate of 58.4 deaths per 1000 total births (95% CI = 40.3–81.4) and an overall perinatal mortality rate 102.2 deaths per 1000 births (95%CI = 79.4–130.6) in the first 7 days of life. The perinatal mortality rate was (48/348) 138 deaths per 1000 total births among referred women and (8/200) 40 deaths per 1000 total births among those with no history of referral. The perinatal mortality rate was (44/331) 132.9 deaths per 1000 total births in the high maternal lactate level group and (12/217) 55.3 deaths per 1000 total births in mothers with normal lactate levels. The details are provided in Table 2.

**Table 2:** Comparison of outcomes in those with and without perinatal deaths among women with obstructed labour

Outcome	Perinatal death [***n*** = 548(%)]	Crude RR	***P***-value
	Yes/No (%)	(95%CI)	
**Mean fetal weight (Kg)**	**–**	**0.95 (0.52–1.71)**	**0.844**
< 2.5	2/14 (14.3)	1.36 (0.36–5.16)	0.660
2.5–3.5	34/335 (61)	1	–
> 3.5	20/143 (14)	1.33 (0.79–2.24)	0.970
**Cord blood lactate at birth, mmol/L**			
**Umbilical artery**^a^	**–**	1.19 (1.15–1.24)	< 0.001
Normal lactate (<=4.8)	1/110 (0.9)	1	–
High lactate (> 4.8)	55/382 (14.4)	13.97 (1.95–100.03)	0.006
**Umbilical vein**^a^	**–**	1.24 (1.18–1.30)	< 0.001
Normal lactate (<=4.8)	1/132 (0.8)	1	–
High lactate (> 4.8)	55/360 (15.3)	17.6 (2.46–126.4)	0.003
**One-minute Apgar Score**			
Normal (> =7)	4/90 (4.4)	1	–
Abnormal (< 7)	52/402 (12.9)	37.17 (13.68–101.01	< 0.001
**Five-minute Apgar score**			
Normal (> =7)	6/481 (1.2)	1	–
Abnormal (< 7)	50/11 (454)	66.53 (29.76–148.75)	< 0.001

^a^ Median and interquartile range (*N* = 490)

### Determinants of perinatal death

The probability of perinatal death was 184% higher among newborns of referred women compared to those with no history of referral, adjusted risk ratio of 2.84 (95% CI 1.35–5.96). The probability of perinatal death was 142% higher among newborns exposed to high maternal blood lactate compared to those exposed to normal maternal blood lactate levels, crude risk ratio of 2.42 (95% CI 1.30–4.45). The reported duration of labour was not associated with perinatal death in this study [Adjusted Risk Ratio, ARR, 0.99, 95% CI (0.97–1.00)]. The details are in Table 3.

**Table 3:** Factors independently associated with perinatal mortality among women with obstructed labour at Mbale regional referral hospital

Outcome	Perinatal death (***n*** = 548)	Crude RR	Adjusted RR
		(95%CI)	(95% CI)
**Maternal venous lactate at base line**			
Normal lactate (<=4.8)	12/205 (5.9)	1	1
High lactate (> 4.8)	44/287 (15.3)	2.40 (1.30–4.45)	2.71 (1.26–4.24)
**Use of traditional medicine**			
Yes	43/272 (15.8)	2.41 (1.33–4.38)	1.80 (0.99–3.27)
No	13/218 (6)	1	1
**History of referral**			
Yes	48/300 (16)	3.45 (1.66–7.15)	2.84 (1.35–5.96)
No	8/192 (4.2)	1	1
**History of rupture of membranes**			
Yes	51/395 (12.9)	2.33 (0.95–5.70)	1.81 (0.75–4.37)
No	5/97 (5.2)	1	1
**Median duration of labour, hours**^a^	28.1 (18.6–28.1)	0.99 (0.97–1.00)	0.99 (0.97–1.00)

^a^ Median (interquartile range)

## Discussion

In this cohort of women with obstructed labour in Mbale hospital, the incidence of perinatal death in the first 7 days of life was very high. The predictors of death were a history of being referred in active labour and having a high maternal blood lactate at diagnosis. Below we discuss these findings in more detail.

### Incidence of perinatal death

The incidence of perinatal mortality in this study was almost four times the national average of 31 per 1000 total births for still births and 27 per 1000 total births for early neonatal deaths, but twice the regional average perinatal mortality of 48 per 1000 total births [16, 17]. This is not surprising because similar findings have been observed elsewhere from facility-based studies. For instance, Kabakyenga et al. reported a perinatal mortality rate of 142 deaths per 1000 live births among women with obstructed labour compared to 65 deaths per 1000 live births among women without obstructed labour from six hospitals in western Uganda [18]. In contrast, reports from community-based studies show lower rates of perinatal mortality because they also include normal women without any obstetric complications known to increase the risk of death. For instance from a cohort of pregnant women enrolled in the PROMISE trial conducted in the greater Mbale district, Nankabirwa et al. reported a perinatal mortality rate of 41 per 1000 pregnancies within the first 7 days of life [22, 28]. Three quarters of the deliveries in this community cohort were complicated, although the complications were not specified, they were probably dominated by obstructed labour. Obstructed labour is characterized by strong and frequent uterine contractions that lead to intrapartum fetal hypoxia which sets a fetus on a pathway to asphyxia, acidosis (metabolic), neuronal injury, long term morbidity or death if the obstruction is not relieved early [4]. This may explain the very high rate of perinatal deaths that we observed in this study. Our estimate of perinatal mortality in this cohort might actually be lower because we excluded women with obstructed labour and confirmed intrauterine fetal death at recruitment. Nevertheless, this information is important because it highlights the need for targeted interventions in the intrapartum period to prevent obstructed labour and mitigate adverse outcomes such as perinatal death in such high risk populations [29].

### Determinants of perinatal death

Newborns to women with obstructed labour and history of being referred were 3 times more likely to die within 7 days of birth compared to those born to participants with no history of being referred. This finding is not very surprising because several studies conducted in low resource settings have identified it as a predictor of adverse maternal and perinatal outcomes [18–20]. Unfortunately, this is probably related to weaknesses in the health care system that contribute to the delays experienced by women in the intrapartum period. In appropriate or delayed actions in the intrapartum period are well known risk factors for adverse maternal and perinatal outcomes [30, 31]. In this setting, referred women usually represent a category of patients with prolonged neglected obstructed labour where the effects of acidosis and asphyxia may not be reversible [13]. Over ¾ (348/477) of the participants in this study were received as referrals from lower government health facilities, which were setup to offer comprehensive emergency obstetric care services as envisioned in the government decentralization policy [32].

In this cohort, we investigated several factors and practices known to increase the risk of perinatal death such as; prolonged duration of labour, the use of herbal medications, and the duration of rupture of membranes [22, 23]. However, none of these was identified as a predictor of perinatal death. This was probably due to the fact that the participants were very similar across factors because they all had comparable exposures as shown in Table 1. We also know that women who experience poorly progress during labour are more likely to consume herbal drugs to try enhance the process. These same patients are also more likely to have prolonged rupture of membranes in the intrapartum period [33, 34]. Additionally, our estimate of duration of labour was not very accurate because it was based on patient reports. Delayed intervention to relieve obstruction is another well-known predictor for adverse outcomes that we could not study in this cohort because every effort was made to ensure that delivery was achieved in less than 120 min for each participant that was randomised [27]. We also had no record of the fluids ingested (both orally and intravenously) since the participants were recruited after a confirmed diagnosis of obstructed labor. So, the study team could not accurately ascertain the interventions instituted in labour and there was no clear and reliable record in the patient’s case files. Although each of them received 1.5 L of normal saline as part of the perioperative care for emergency caserean section as per the current national standard of care for patients with obstructed labour [35].

Newborns to women with obstructed labour and high levels of blood lactate had 2.4 times the risk of dying by the 7th day of life compared to those with normal levels. Previous studies including a recent systematic review, have focused on umbilical blood and amniotic fluid lactate level as a predictor of adverse neonatal outcomes [5, 16, 17]. This is among the first studies to show that maternal venous blood lactate could independently predict adverse neonatal outcomes among women with obstructed labour, irrespective of the duration of labour. This information is important as it could help health workers to prepare for adverse neonatal outcomes such as the need for neonatal resuscitation. In areas with lower cadre primary birth attendants, this information could also be useful in the decision to refer for specialized care or to summon more skilled providers. More so, we used a low-cost, simple point of care device which could easily be operated by lower cadre health workers in rural areas. In low resource settings, the partogram has not been fully embraced by health providers for various reasons that have been documented previously [36]. So, the prospect of including lactate measurement using a point of care device as an add on to the current package of components for intrapartum care is exciting because it could potentially improve the outcomes of pregnancy especially for the newborns. Measurement of maternal lactate offers the extra advantage of early prediction of perinatal outcomes compared to when it is done on umbilical cord blood. Measurements obtained from point of care devices have been shown to correlate well with other measures of acidosis (pH, base excess and blood gases), and it is already in use in several well-resourced settings.

In our study, we used a cut off of 4.8 mmol/L for acidosis which has only been validated for fetal scalp monitoring because we could not find any other published and widely accepted cutoffs for maternal venous lactate in labour. Nordstrom et al. reported a mean maternal venous lactate of 2.6 ± 1.0 (± S.D) mmol/L at the end of second stage of normal labour and also demonstrated an increase every 15 min of bearing down up to 4.3 ± 0.9 mmol/L at 75 min [37]. In this cohort of women with obstructed labour, the median maternal venous lactate level was much higher 6.6 mmol/L and the majority 60% (331/548) were acidotic (≥ 4.8 mmol/L). We suggest that the same cut off could be used for maternal venous blood, pending further studies to establish a cut off for acidosis in this population of patients.

### Methodological considerations

Our choice of a hard-primary outcome for this study is a strength. In addition to the fact that this is may be the first report of the association between high maternal lactate level and perinatal death among patients with obstructed labour. However, we think these were some of the limitations in this study. We did not measure potential confounders such as the duration of rupture of membranes and the decision to incision interval which are known risk factors for obstructed labour. The perinatal mortality could have been much higher if it was not in a setting of a clinical trial where every effort was made to ensure that each of the enrolled women was delivered in under 2 h from diagnosis. Nonetheless, we believe that our findings can be generalized to other regions in Uganda and beyond where there are similar challenges.

## Conclusion

The incidence of perinatal death among women with obstructed labour was very high. The determinants of death within the first 7 days of life were being referred in active labour and having high maternal blood lactate levels.

## Data Availability

The datasets used and/or analysed during the current study are available from the corresponding author on reasonable request.

## Abbreviations

OL: Obstructed labour
FSB: Fresh still birth
ENND: Early neonatal death
CI: Confidence interval
RR: Risk ratio
ARR: Adjusted risk ratio
PD: Perinatal death
SDG: Sustainable development goal
ENAP: Every newborn action plan
UDHS: Uganda demographic and health survey
ANC: Antenatal care
